# Centre- versus home-based exercise among people with mci and mild dementia: study protocol for a randomized parallel-group trial

**DOI:** 10.1186/s12877-017-0684-0

**Published:** 2018-01-25

**Authors:** Laura E. Middleton, Sandra E. Black, Nathan Herrmann, Paul I. Oh, Kayla Regan, Krista L. Lanctot

**Affiliations:** 1 University of Waterloo, 200 University Ave W (BMH 1114), Waterloo, ON N2L 3G1 Canada; 2 Sunnybrook Health Sciences Centre, 2075 Bayview Ave, Toronto, ON M4N 3M5 Canada; 3 University Health Network, 347 Rumsey Rd, Toronto, ON M4G 2V6 Canada; 4 Sunnybrook Research Institute, 2075 Bayview Ave, Toronto, ON M4N 3M5 Canada

**Keywords:** Exercise, Mild cognitive impairment, Dementia, Delivery of health care, Clinical trial

## Abstract

**Background:**

Worldwide, almost 50million people lived with dementia in 2016. A cure or disease modifying pharmaceutical treatment for dementia remains elusive so alternative therapies are of critical importance. Mounting evidence supports exercise in the prevention and therapy of dementia. However, the cognitive, physical, and psychological challenges common to dementia along with a poor understanding and accommodation of dementia in the community are major barriers to exercise. Consequently, effective delivery options need to be identified. The primary objective of this study is to compare the effectiveness of center-based (CB) exercise versus home-based (HB) exercise for achievement of physical activity guidelines among people with MCI or mild dementia.

**Methods:**

This is a randomized parallel-group trial comparing the effects of CB and HB exercise adherence among community-dwelling adults ≥50 years with a clinical diagnosis of MCI or mild dementia. Participants will be randomized to either CB or HB exercise. The CB group will meet weekly for small group exercise and will be prescribed additional exercise to be completed independently. Participants in the HB group will be given a physical activity prescription to be completed independently in the community. Participants in HB will also be contacted by phone monthly to adjust exercise prescriptions. The primary outcome will be achievement of exercise guidelines (150 min/wk. of moderate activity) assessed using an activity monitor. Secondary objectives will evaluate cost-effectiveness and the influence of individual and environmental factors on the primary outcome. Tertiary outcomes include physical function, cognition, mood, and quality of life.

**Discussion:**

There is scant research to indicate the most effective way to deliver exercise to people with MCI and mild dementia, which is needed specifically because these groups face significant barriers to exercise. To capitalize on the benefits of exercise, feasible exercise delivery options need to be identified. The results of this study will directly complement ongoing clinical trials and will be essential to implementing exercise recommendations specific to the prevention and therapy of dementia in a feasible and cost-effective manner when they emerge.

**Trial registration.:**

Clinicatrials.gov; Identifier: NCT02774720 (version updated December 12, 2016).

## Background

Worldwide, there were 46.8 million people living with dementia in 2016. [1] This number is expected to reach 131.5 million by 2050. [1] Since there is currently no disease modifying agent or cure for dementia, it is essential to identify alternate preventative and therapeutic approaches. Exercise is one promising strategy to prevent and treat dementia. [2] Convergent evidence from cohort studies, clinical trials, and animal models supports exercise as an intervention to improve cognitive function among older adults with and without cognitive impairment, [3] though more rigorous randomized controlled trials are still needed. [4, 5]

There is no doubt that exercise has benefits, however, for older adults with and without cognitive impairment. Exercise is recommended by the American College of Sports Medicine for older adults to improve health, functional capacity, quality of life, and functional independence. [6] A meta-analysis of exercise trials among people with cognitive impairment confirmed that exercise programs as short as 3 months improved daily function and physical function among this group. [7] Specifically, exercise improves daily function, mobility, and balance among those at risk for (with mild cognitive impairment (MCI)) or with dementia. [7–10] Accordingly, the Ontario Brain Institute (CANADA) developed consensus recommendations of 150 min per week of moderate aerobic exercise and twice weekly strength training for the prevention and therapy of dementia, [11] in line with general guidelines for older adults in many countries (for example, [12–14]).

Even with the substantial associated benefits, many older adults remain inactive, [15, 16] and people with mild cognitive impairment (MCI) and dementia are more sedentary than their peers. [17, 18] Persons with dementia and their care partners report that the cognitive, physical, and psychological challenges common to dementia (e.g., memory and attentional deficits, apathy, co-morbid health problems [19, 20]). In addition, persons with dementia typically experience a loss of freedom as cognitive impairment progresses, which means that persons with dementia may be dependent on their care partner (or others) to access both local and more distant opportunities. [21] This means that care partner support for exercise in addition to the health of and fatigue among their partners has a strong influence exercise behaviours. [21] Furthermore, a poor understanding and accommodation of dementia in the community may lead to explicit or implicit exclusion of persons with dementia from exercise programs, further reducing opportunities. [21]

Despite substantial support for exercise as a therapy for people with MCI or dementia, we have a poor understanding of the best strategies to increase exercise levels in this group. [22] Clinical trials of community-dwelling adults with MCI or dementia have primarily, [5] but not always, [23, 24] employed highly controlled, centre-based interventions which increase internal validity but poorly reflect what is available in the community. Prior comparisons of center-based [CB] versus home-based [HB] exercise programs among various patient groups and populations indicate that the relative effectiveness varies by group,[25] suggesting a need to study the effectiveness of these delivery options in unique patient groups. Moreover, a recent review specifically highlighted the need to identify effective exercise delivery options specific to people with MCI and dementia. [22]

Trials of exercise interventions among people with MCI or dementia indicate that both CB and HB exercise can be effective for improving cognitive, physical, and other outcomes (for example, [23, 26]). It is possible that the most effective option will not be uniform even within this clinical group but may depend further on the individual (e.g., degree of care partner support, diagnosis (i.e. MCI vs dementia), severity of deficits) and the context (e.g., weather, proximity to facilities). Consequently, the aim of this study is to compare the effectiveness of CB versus HB exercise programs in achieving aerobic exercise guidelines (percent of 150 min per week of moderate intensity activity) among people with MCI and mild dementia. Secondary objectives will compare the cost-effectiveness of the interventions and examine the influence of individual and environmental factors on achievement of the primary outcome. Exploratory objectives will compare the effect of CB and HB exercise on physical function, cognitive function, vascular health, quality of life, instrumental activities of daily living (IADLs), mood, and individual goal attainment. Based on prior results among healthy older adults, [27] we hypothesize that participants assigned to the HB exercise group will have a higher percent achievement of physical activity guidelines compared to the CB exercise group. Conversely, we hypothesize that the CB group will experience greater gains in physical function than the HB group.

## Methods & design

This is a pragmatic, randomized, parallel group trial to explore the relative effectiveness of two exercise delivery options (CB versus HB) among 60 people with MCI and mild dementia. The study will be conducted at two sites in Canada: Toronto (Sunnybrook Health Sciences Centre and University Health Network-Toronto Rehabilitation Institute) and Kitchener-Waterloo (University of Waterloo). Enrollment of participants began in September 2016 and is expected to continue until September 2018.

**Fig. 1:**
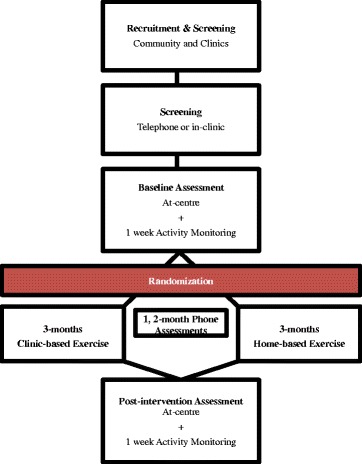
Flow of study participants

The study flow is shown in Fig. 1. Assessments will occur at baseline and immediately after the completion of the intervention. In addition to these assessments, physical activity, health status, and health care utilization will be reported monthly. Participants will be randomly assigned to CB or HB after baseline assessments, blocked by site, via a central internet randomization service (www.randomization.com). The randomization will be managed by a department research assistant not involved in the assessments and communicated to the participants directly or via the site research coordinator. Those who assess and those who analyze outcomes will remain blinded to allocation throughout and will report whether there was any indication of group allocation. In addition, analyses will be done by code (that is, Group ‘A’ and ‘B’) with code only revealed after analyses are complete. Due to the nature of the intervention, participants and exercise physiologists will not be blinded to group but participants will be blinded to the study hypotheses.

This study has been approved by an Ethics Committee for Human Research at the University of Waterloo, Kitchener-Waterloo Tri-Hospitals, Sunnybrook Health Sciences Centre, and the University Health Network. This study has been registered at *clinicaltrials.gov* with the identifier NCT02774720 (version updated December 12, 2016).

### Participants

We will recruit people with MCI or mild dementia from both clinics and the community. Participants will be recruited from memory and geriatric clinics as well as through flyers distributed through local community centres and groups, such as the Alzheimer Society and dementia support groups. Promotion through news articles and/or advertisements will be used to supplement recruitment, where necessary. Interested individuals will be screened either in the clinic or by phone using inclusion/exclusion criteria and the Canadian Society of Exercise Physiology physical activity readiness questionnaire-plus (PAR-Q+), [27] a screen for physical activity readiness that reviews common counter-indications to exercise. A consent and screening session will be arranged for those who appear eligible and are interested.

Inclusion and exclusion criteria are purposely broad to reflect the spectrum of people with MCI and mild dementia treated in academic and community clinics. To be eligible for the study, people must: 1) have a clinical diagnosis of MCI or dementia, as determined by the treating physician as opposed to specific criteria; 2) MMSE > 22 or MoCA > 17; 3) be 50 years or older; 4) have a stable pharmaceutical regimen for at least 2 months; 5) be able to travel to the CB site; 6) have a care partner who will support at-home exercise and provide information for some assessments; 7) be able to complete a 2-min walk; 8) have adequate English to understand exercise training and assessments; 9) have adequate hearing and vision for cognitive tests; 10) be able to comply with assessment and training schedule; and 11) be screened safe for exercise by either a physician or certified exercise physiologist. People who currently participate in moderate exercise ≥3×/week or have cardiovascular, musculoskeletal, pain, or other co-morbid conditions that preclude exercise will be excluded.

### Interventions

We will use a pragmatic approach in this trial. That is, the interventions are meant to reflect exercise delivery options that could realistically be implemented in clinics and the community. The primary advantage of this approach is that its results are more likely to translate to the ‘real-world’. Both interventions are designed to help participants reach exercise recommendations for the prevention and therapy of dementia (≥150 min/week of moderate aerobic exercise with resistance training 2×/week) by the end of the intervention period. [11] The exercise prescriptions for participants in both groups will be individualized in consideration of the participant’s baseline exercise, fitness, and mobility and will progress gradually to meet targets.

#### Centre-based exercise

A certified exercise physiologist will lead 60 min exercise sessions that include both aerobic, resistance, balance, and flexibility training at the centre once per week in a small group setting (3 to 5 participants), which is a frequency and duration often available in clinical and community exercise programs. Treadmill or Nustep will be the main aerobic training modalities during in-class sessions. Intensity will be monitored by heart rate and rating of perceived exertion (RPE, target: 12–15/20). Resistance training will target major muscle groups using exercise machines and hand-held weights as well as through functional activities. Exercise classes at the centre will be complemented by additional exercise to be performed independently at-home or in the community. The exercise physiologist will prescribe at-home exercise and collaborate with the participant to develop a plan for meeting the prescription. Participants will be encouraged to invite their care partner to attend this session. Participants will use an exercise diary to record their daily exercise.

#### Home-based exercise

The HB program replicates an intervention used previously among middle to older aged adults, [28] except that care partners will be involved in the program planning. As with the at-home portion of the CB group, the exercise physiologist will prescribe exercises to be performed independently at-home or in the community and will develop a plan with the participant and care partner for meeting the prescription. The participant will mail in their exercise diary monthly. In addition, the exercise physiologist will talk to the participant and care partner each month by phone to discuss achievements and strategies to overcome recent barriers. Following the call, the exercise physiologist will adjust the exercise prescription and mail it to the participant with instructions to contact if there are any questions.

### Measures

All participants will be assessed at the center at baseline and after the intervention. Participants will also complete a telephone assessment 1 month and 2 months into the program. Measures by time points are listed in Table 1. Assessors are blinded to group allocation throughout the study. Any possible disclosure will be noted.

**Table 1:** Assessments by time point

	Baseline	1-month	2-month	Post-intervention
Participant Descriptors				
Participant information questionnaire	X			X
Height, weight, waist, BP	X			X
Medications	X			X
Physical activity history	X			
Social Support for exercise survey	X			X
BBAQ	X			X
Self-reported health	X	X	X	X
Falls	X	X	X	X
Secondary & Tertiary Outcomes				
RUD-Lite	X	X	X	X
PASE	X	X	X	X
ADAS-Cog	X			X
Stroop Test	X			X
Trails Making Task	X			X
Semantic Fluency	X			X
SPPB	X			X
DAD-6	X			X
EQ-5D	X			X
NPI-Q	X			X
6 min walk	X			X
25 ft. single & dual task	X			X
GAS	X			X
Primary Outcome				
Actigraph	1-week following			1-week following

*BP* blood pressure, *BBAQ* Barriers to Being Active Questionnaire, *RUD-Lite* Resource Utilization Dementia-Lite questionnaire, *PASE* Physical Activity Scale for the Elderly, *ADAS-Cog* Alzheimer Disease Assessment Scale-cognitive, *SPPB* Short Physical Performance Battery, *DAD-6* 6-item Disability Assessment for Dementia scale, *EQ-5D* Euro-Qol 5D, *NPI-Q* Neuropsychological Inventory, *GAS* Goal Attainment Scaling

#### Primary outcome

The primary outcome will be achievement of aerobic activity guidelines for older adults (percent of 150 min/week of moderate physical activity) as measured using an activity monitor (Actigraph GTX3+), [29] which contains an accelerometer that is validated for measurement of physical activity by intensity among older adults. [30, 31]. Participants will wear the activity monitor for one week following the baseline assessment, with the expectation that at least 5 days of usable data will be achieved as is required for reliable estimates of habitual physical activity. [32] Wear time will be validated by established parameters. [33] We will determine time spent in moderate or vigorous physical activity using cut-offs based on recent studies of older adults (≥810 cpm). [35] The time spent in moderate or vigorous activity will be divided by the recommendation (150 min/week or 21.4 min/day) to get percent achievement of the guidelines.

#### Secondary and tertiary outcomes

*Cost-effectiveness* will be determined by cost per unit change in physical activity (min/wk. of moderate physical activity). Participants will report health-care utilization each month, including visits to health care professionals, admissions or visits to the hospital, and laboratory work using the Resource Utilization Dementia (RUD)-Lite questionnaire, as employed in previous studies regarding exercise in cognitively impaired populations. [34, 35] Costs will then be assigned to health-resource utilization data (using a Canadian healthcare system perspective) and combined with the costs for each intervention group to generate a cost per min/week of exercise.

*Physical Activity* will also be reported at baseline, monthly during the intervention, and post-intervention using the Physical Activity Scale for the Elderly (PASE). The PASE is valid among older adults aged 65–100 years and for proxy responses by care partners. The PASE will be used to track physical activity change by month over the course of the intervention, to better understand the change in physical activity over time. In addition, it will be used to assess achievement of resistance training guidelines (2×/wk). [36]

*Cognitive Function* will be assessed with three measures. The Alzheimer Disease Assessment Scale-cognitive (ADAS-Cog) will be used to assess global cognitive function. [37] The ADAS-Cog is considered sensitive to the earliest cognitive changes in MCI and has been sensitive to exercise interventions. [24, 38] The Stroop Test will be used to measure executive function (inhibition), [39] which is arguably the cognitive domain most sensitive to exercise effects. [40] The Trail Making Task and Semantic Fluency will additionally be used to test additional components of executive function and language. [41, 42]

*Physical function* will be assessed using a 6-min walk test, the Short Physical Performance Battery, and single and dual-task gait. [43, 44] The 6-min walk is a functional measure of aerobic fitness that has a reliability of 0.95 among older adults. [44] The Short Physical Performance Battery includes measures of functional strength (chair stands), balance (semi-tandem, side-by-side, tandem stand), and walking speed (4 m walk) and is associated with functional disability, mortality, and institutionalization among older adults. [43] Gait speed will also be measured over 25 ft. Participants will complete a single task gait speed assessment, where they will walk the 25 ft. course at their usual walking speed. Participants will also complete a dual task gait speed assessment where they will walk the 25 ft. course at their usual walking speed, while simultaneously reciting words aloud that begin with a specified letter of the alphabet.

*Daily function* will be assessed with the 6-item Disability Assessment for Dementia scale (DAD-6), [45] .which is intended to detect early changes in activities of daily life in people with cognitive impairment. [45]

*Apathy and depressive symptoms* will be assessed using the Neuropsychological Inventory, which is a valid and reliable tool for the assessment of neuropsychiatric symptoms in people with cognitive impairment. [46]

*Health-Related Quality of Life* will be measured using the EuroQol 5D (version EQ-5D-3 L), [47] which had the best convergent validity among self- and proxy-ratings for people with cognitive impairment. [48] The EuroQol 5D can also generate utility measurements for different health states, which can be employed for cost-utility analyses.

*Physical Health* will be characterized using height, weight, waist circumference, and blood pressure. We will use this data to create simple indicators of vascular risk (hypertension, obesity).

*Goal attainment* of individually-important goals will be measured using goal attainment scaling. [49, 50] Goal attainment scaling is a feasible, valid, and reliable method of goal setting and measurement in older adults. [49, 50] At least three individually-important goals will identified by the participant. The expected outcome (score of 0) will be no change from the current state. Criteria for better or much better than expected and for less than and much less than expected will then be determined jointly between the individual and the exercise physiologist. At the post-intervention assessment, each attainment of each goal will be scored between −2 and +2.

#### Individual characteristics

Participants will report demographic information, education, medications, and co-morbid conditions at baseline. Each participant will have height, weight, waist circumference, and blood pressure measured. In addition, participants will provide self-reported health (poor to excellent) at baseline, monthly, and post-intervention. Participants will also report their history of injury, surgery, and major diseases as well as current health conditions and medications. Use of a mobility aid and recent falls will be reported.

Exercise in the teenage years and mid-life will be reported as history of participation in strenuous exercise 3×/week (Y/N as done previously). [51] Barriers to exercise will be reported using the Barriers to Being Active Quiz, [52] where individuals report how likely they are to say specific statements regarding barriers to exercise. The barriers to exercise are then summarized into six categories: time, social influence, lack of energy, lack of willpower, fear of injury, lack of skill, and lack of resources.

Care partner support for exercise will be gathered using a social support for exercise survey, [53] adapted specifically for care partners.

#### Environmental characteristics

Address will be recorded in order to determine the walkability of the neighborhood, as characterized by Walk Score (www.walkscore.com). The Walk Score is an indicator of sidewalks and neighborhood amenities. Weather, including precipitation and high temperature, will be recorded from Weather Canada reports.

### Sample size calculation

Sample size is based on the primary objective, to detect a significant difference in percent achievement of physical activity recommendations (continuous variable) between CB and HB groups over the intervention period. A recent Cochrane review of CB versus HB among older adults indicated that HB exercise had better physical activity achievement than CB, with an effect size of 0.8. [27] There were no studies that targeted people with cognitive impairment. To account for the possibility that cognitive impairment may reduce the feasibility of HB, we used a smaller effect size of 0.6, an alpha of 0.05 and a beta of 0.85 with two-group repeated measures MANOVA (within and between factors) to estimate a sample size of 39. We assumed 35% drop out, similar to our pilot work and to a previous study of exercise in people with MCI, [54] to bring the required sample size to 60.

### Analysis

Reporting will be according to CONSORT criteria. Analyses will follow the intention-to-treat principle. Missing data will be treated using multiple imputation, as recommended for people with dementia. [55]

#### Primary objective

Mixed effects linear regression will be used to analyze differences in the primary outcome (percent achievement of physical activity recommendations) as a function of group (CB, HB), time (baseline, follow-up and interim month 1, month 2 where relevant), and group x time. A random effect for participants and site (CB) will be included in the models.

#### Secondary objectives

*(i) Cost-effectiveness:* We will use a Canadian healthcare system perspective in our cost-effectiveness analysis, with a 6-month time horizon. Costs will be assigned to health-resource utilization data for each exercise group and combined with data on cost per participant for the program. We will then sum costs of each intervention group and the costs of health-care utilization for each unit change in physical activity (min/wk. of moderate physical activity) to generate a cost per min/week of exercise. We anticipate that HB will be economically attractive. *(ii) Influence of person and setting-specific factors:* Association of person- and setting-specific characteristics with the primary outcome with be examined using repeated measures mixed effects linear regression but with the characteristic included as a factor in the model.

#### Tertiary objective

Change in each tertiary outcomes between T1 and T2 will be examined using mixed effects linear regression as a function of group, time, and group x time interaction. A random effect for participants and site will be included in the models.

## Discussion

This study will examine the effects of CB versus HB exercise on physical activity as well as cost, cognitive function, physical function and health, functional ability, apathy and depressive symptoms, and quality of life among people diagnosed with MCI or mild dementia. We will consider whether outcomes or the relative effectiveness of the interventions varies according to individual or environmental factors.

The prevalence of dementia worldwide is expected to nearly triple in the next 35 years. [1] Increasing research suggests that exercise can improve cognitive function, physical function, as well as functional abilities among people with MCI and dementia. [5] Despite these benefits, people with MCI and dementia are frequently inactive. [17, 18] Effective strategies to increase exercise in this group have not been identified and are needed to leverage exercise-related benefits. [22] Most often, exercise trials have used either highly controlled interventions, which are not reflective of community exercise opportunities, or occur within residential care. [5] Neither offers insight into effective strategies for community-dwelling adults with cognitive impairment. People with cognitive impairment have several differences (cognitive function, physical function, mood, mobility) that may alter the feasibility of exercise delivery options compared to older adults more generally and may make some programming options less accessible. [19–21, 56, 57] This study takes a pragmatic approach to evaluating the effectiveness of exercise delivery options as could be available in the community among people with MCI or mild dementia to understand how well, in whom, and where each option will work in ‘real-life’.

The results from this trial will directly complement ongoing clinical trials regarding the efficacy of exercise to help inform implementation of emerging exercise guidelines for the prevention and therapy of dementia in a feasible and cost-effective manner. Prior studies comparing HB versus CB exercise among other populations differ in findings, suggesting the relative effectiveness may be population specific. Furthermore, it is likely that both exercise levels and the optimal method of exercise delivery will differ according to the individual’s characteristics, experiences, and circumstances as well as the environment in which they live. For example, the care partner for a person with dementia may be a primary influencer of the person’s exercise level, whether it be a positive or negative influence. Often, people with MCI or dementia will rely on their care partner for travel to exercise facilities so partners who are unsupportive or in poor health can restrict exercise opportunities outside of the home (CB exercise). In contrast, CB exercise may be more appealing if travel distances are short, so that the benefits of guided exercise outweigh the downsides of time and effort.

Study limitations should also be acknowledged. We purposely include individuals with a range of cognitive impairment and a variety of causes in order to best represent the population seen in clinical practice. However, if significant variability exists in the influence of interventions, then Type II error may occur – a lack of significant effect in our sample despite a true effect in the population. However, including a representative sample increases the generalizability of our results. In addition, we take a pragmatic approach in this study and include interventions as could be delivered in practice. This has the advantage of translating readily to ‘real-life’. The disadvantage is that the magnitude of effects may be smaller than with highly controlled interventions and the cause of differences is more difficult to determine.

No cure or even disease modifying therapy for dementia, and particularly Alzheimer’s disease, appears to be on the immediate horizon. [58] Consequently, non-pharmacological strategies for prevention and therapy of dementia are increasingly recognized as critical to the management of dementia risk and progression. Exercise is one of the most promising strategies to not only improve cognitive function and reduce the risk for dementia but also to improve physical function, functional independence, and quality of life among people with cognitive impairment. Even if exercise does not yield cognitive benefits, it is likely that the improvements in physical function will improve functional abilities, which could delay dementia diagnosis. [59] Results from our study will help inform the implementation of effective exercise delivery strategies to enable people with MCI and dementia to capitalize on the extensive benefits of exercise.

## Abbreviations

CB: Centre-based (exercise)
HB: Home-based (exercise)
MCI: Mild cognitive impairment
MMSE: Mini-mental state examination
MoCA: Montreal Cognitive Assessment
